# Addition of nisin to high-viscosity glass-ionomer cement: a comparative in vitro study on antibacterial and physical properties

**DOI:** 10.1007/s40368-024-00910-w

**Published:** 2024-05-14

**Authors:** D. Hegde, B. S. Suprabha, K. Ginjupalli, E. Suman, S. Natarajan, R. Shenoy, A. Rao

**Affiliations:** 1 Department of Pediatric and Preventive Dentistry, Manipal College of Dental Sciences Mangalore, Manipal Academy of Higher Education, Manipal, Karnataka India; 2 Department of Dental Materials, Manipal College of Dental Sciences Manipal, Manipal Academy of Higher Education, Manipal, Karnataka India; 3 Department of Microbiology, Kasturba Medical College Mangalore, Manipal Academy of Higher Education, Manipal, Karnataka India; 4 Department of Oral Pathology and Microbiology, Manipal College of Dental Sciences Mangalore, Manipal Academy of Higher Education, Manipal, Karnataka India; 5 Department of Public Health Dentistry, Manipal College of Dental Sciences Mangalore, Manipal Academy of Higher Education, Manipal, Karnataka India

**Keywords:** Nisin, Glass ionomer cement, Antibacterial agents, Physical phenomena

## Abstract

**Purpose:**

Nisin is a lantibiotic effective against Gram-positive microorganisms such as *Streptococcus mutans*. The study aimed to determine the effect of the addition of nisin to high-viscosity glass-ionomer cement (HVGIC) on its antibacterial activity, setting time, surface microhardness, and compressive strength.

**Methods:**

1 and 3% w/w nisin were added to HVGIC before mixing. Unmodified HVGIC was the control. Agar disc diffusion, direct contact test, and scanning electron microscopy (SEM) analysis were used to evaluate antibacterial activity against *S. mutans*. Setting time, surface microhardness, and compressive strength were measured using Gilmore needle apparatus, digital microhardness tester, and universal testing machine, respectively. Statistical analysis included Student’s t test, one-way ANOVA with Tamhane’s post hoc test, and repeated-measures ANOVA.

**Results:**

As evidenced by the agar disc diffusion (*p < *0.001), direct contact tests (*p = *0.025), and SEM analysis of the *S. mutans* cell count and cell surface area (*p = *0.049 and 0.003), 3% nisin had the strongest antibacterial activity. There was a dose-dependent increase in setting time (*p = *0.005) and surface microhardness (*p = *0.006), with no significant difference in compressive strength compared to control.

**Conclusion:**

The addition of 3% nisin to HVGIC enhances the antibacterial action against *S. mutans* and surface microhardness without adversely affecting setting time and compressive strength.

## Introduction

Atraumatic Restorative Treatment (ART) is a minimally invasive method of managing cavitated lesions where only hand instruments are used for caries removal, and the cavity is restored with an adhesive restorative material such as high-viscosity glass-ionomer cement (HVGIC) (Saber et al. 2019). The antibacterial activity is vital for HVGIC used with the ART technique, as residual caries remain after hand excavation (Bönecker et al. 2003). In addition, the antibacterial surface properties of the material are essential to prevent biofilm formation on the restoration and, thus, marginal caries (Davidovich et al*.* 2007). The glass-ionomer cement's (GIC) caries-preventing ability is attributed to its antibacterial activity due to fluoride release and low pH during setting (Vermeersch et al. 2005). However, the antibacterial activity decreases upon setting (Davidovich et al. 2007; Wiegand et al. 2007). Clinical study data show that GIC restorations do not entirely inhibit secondary caries (Wiegand et al. 2007; Ge et al. 2023). Therefore, there is a need to enhance to antibacterial properties of HVGIC (Chen et al. 2020). The incorporation of antimicrobial additives like chlorhexidine, triclosan, cetylpyridinium chloride, benzalkonium chloride, and antibiotic combinations which inhibit cariogenic microorganisms such as *Streptococcus mutans* have been studied earlier but have been known to affect the physical properties of the cement (Yesilyurt et al. 2009; Prabhakar et al. 2013).

Nisin is an antimicrobial peptide or bacteriocin produced by *Lactococcus lactis* that is classified as a type A lantibiotic due to lanthionine and B-methyl lanthionine amino acids in its structure (Cotter et al. 2005; Willey et al. 2007). It is an odourless, colourless, tasteless substance with no cytotoxicity against human cells (Shin et al. 2015; Chan et al. 2023). It is licensed in many countries worldwide as a safe antibacterial agent in cured meat, dairy, canned, and plant protein foods (Cotter et al. 2005; López-Cuellar et al. 2016.). It inhibits the growth of Gram-positive organisms, including cariogenic bacteria such as *S. mutans,* and multi-species biofilm formation at concentrations as low as 1 µg/ml (Shin et al. 2015). Salivary constituents do not hamper the antimicrobial activity, and unlike conventional antibiotics, no drug resistance is developed (Tong et al. 2010; Shin et al. 2015). Recent in vitro studies incorporating nisin into total-etch and universal adhesives inhibited *S. mutans* biofilm formation without affecting bonding properties (Su et al. 2018; Zhao et al. 2020; Keerthipriya et al. 2021; Panpisut et al. 2021).

However, the effects of the addition of nisin to GIC are yet to be studied. The incorporation of nisin into HVGIC has the potential to enhance its antibacterial activity without affecting its physical properties. This study aimed to assess and compare the antibacterial activity against *S. mutans*, net setting time, surface microhardness, and compressive strength of nisin-modified HVGIC with unmodified HVGIC. The null hypotheses were that there is no difference in the diameter of the inhibition zone, optical density values (at all time points), *S. mutans* count, net setting time, surface hardness, and compressive strength between 1% nisin-modified HVGIC, 3% nisin-modified HVGIC, and conventional HVGIC.

## Materials and methods

The current in vitro study began after obtaining clearance from the Institutional Ethics Committee, Manipal College of Dental Sciences Mangalore (Protocol Reference Number: 20072).

*Sample size:* It was estimated assuming a relative difference of 30% in the antibacterial activity (Matalon et al. 2009) and surface microhardness (Chen et al. 2020) between the unmodified HVGIC and the nisin-modified HVGIC, using ClinCalc.com (Calculator and ClinCalc 2019). At 95% confidence interval and 80% power, each group's sample size was calculated to be 9. Thus, the sample size for the antibacterial activity tests (agar disc diffusion method and direct contact test) and surface microhardness was 9 in each group.

The sample size for assessing net setting time and compressive strength was determined according to the ANSI/ADA Standard No. 96 (which is a modified adoption of ISO 9917:1991) for dental water-based cements (*n* = 3 and *n* = 5, respectively).

*The groups included in the study were:* Commercially available, unmodified HVGIC (GC Fuji IX Gold Label, GC Corporation Tokyo, Japan) was the control group (C). The test groups were Group 1(G1) and Group 2 (G2), and nisin was added at concentrations of 1% and 3% (w/w), respectively.

*Sample preparation:* All weight measurements were done using a micro-measuring balance (SAB 103L Scaletec, Pune, India) with a measuring accuracy of 0.001 g. The weight of the mixing paper pad was deducted during measurements. One drop of the HVGIC liquid (GC Fuji IX Gold Label, GC Corporation Tokyo, Japan) was dispensed onto the pad and weighed. For every 1 g HVGIC liquid, 4.6 mg and 13.8 mg nisin powder (2.5%w/w pure) (Sigma-Aldrich, St Louis, USA) was added to obtain 1% and 3% (w/w) concentrations for Groups 1 and 2, respectively. The nisin powder was mixed into the HVGIC liquid just before each sample preparation using a plastic spatula until a clear liquid was obtained. Premeasured HVGIC powder (GC Fuji IX Gold Label, GC Corporation Tokyo, Japan) was added to the nisin-containing liquid using a plastic spatula, maintaining a powder–liquid ratio (p/l) of 3.6:1(w/w), following the manufacturer’s instructions, in two equal parts at room temperature. The mix was then packed into petroleum jelly-coated moulds, whose dimensions followed the requirement of each experiment, to obtain test samples. For the control group (Group C), liquid without nisin was used and was mixed per the manufacturer's instructions and packed into the moulds in the same manner as the test groups. The moulds were packed in incremental layers to avoid voids. The specimens were then covered with polyester sheets (Ecodent, Gurgaon, India), flattened with a microscopic glass slide, and allowed to set for one hour. They were finished with 400-grade silicon carbide paper under constant water irrigation. The dimensions of the specimens were verified with Vernier callipers, coated with petroleum jelly for surface protection, and stored in Borosil® glass containers (Borosil Limited, Mumbai, India) with distilled water for 24 h before testing for all physical properties. The specimens for antibacterial activity tests were sterilised for 12 h in the UV Light Chamber (Sentinal Gold, ESCO Singapore).

### Antibacterial activity

*Agar disc diffusion test (ADD)* It measures the cement's antibacterial activity based on the solubility and diffusability of the antibacterial component (Matalon et al. 2009). Nine discs per group were prepared using a 6 mm-depth and 4 mm-diameter brass circular disc-shaped mould. All microbiological experiments were performed in the Biosafety level II (BSL II) lab, using ESCO class II type A biosafety cabinet, maintaining sterile conditions. A loopful of the lyophilised *S. mutans* (MTCC 497, Microbial Type Culture Collection and Gene Bank, Chandigarh, India) was transferred to 5 ml Brain Heart Infusion (BHI) broth with 0.5% bacitracin, incubated for 24 h in a 5% CO_2_ incubator (Nuaire, Plymouth USA) at 37 ± 0.5 °C. The bacterial growth was adjusted to 0.5 Mcfarland’s standard [10^8^ colony forming units (CFU) per ml]. A lawn culture of *S. mutans* was prepared by swabbing the surface BHI agar supplemented with 5% sheep blood in a 4 mm-depth Petri dish. The set disc-shaped specimens were placed on the medium, maintaining a 24 mm distance between the two discs. The plates were then incubated at 37 ± 0.5 °C for 48 h using a 5% CO_2_ incubator (Nuaire, Plymouth, USA). The diameter of inhibition zones around the specimens was measured using Vernier Callipers at three different points, and an average value was obtained at 24 and 48 h. The absence of bacterial growth in the halo region was confirmed under the light microscope at 10× magnification. All measurements were done by two independent examiners blinded to the group type.

*Direct contact test (DCT)* evaluates the effects of direct and close contact between the test microorganism and the substance under test (Matalon et al. 2009). Using a flat-ended instrument, the bottom of the 9 wells of a 96-well round-bottomed microtiter plate (HiMedia Pvt Ltd, Mumbai, India) was equally coated with the cement mix from each group. All the samples were then sterilised for 4 h using UV Light Chamber (Sentinel Gold, ESCO Singapore). The surface of each sample was then covered with 10 μl of *S. mutans* suspension (10^8^ CFU/ml) and incubated at 37 °C for 1 h. Further, 250 ml of BHI broth containing 0.5% bacitracin was added to each well after the suspension liquid had evaporated for direct contact between the bacteria and the test material's surface. The identical inoculums placed at the bottom of four microwells not coated with the cement formed the positive control (PC). The uninoculated medium added to four microwells coated with the cement at the bottom formed the negative control (NC). The microtiter plate was then incubated at 37 ± 0.5 °C in a 5% CO_2_ incubator. The Optical Density values (OD) were recorded spectrophotometrically (ELISA Reader ELX-800, Biotek Vermont USA) at 650 nm at 3, 6, and 24 h after inoculation of the broth to measure the bacterial growth. Test samples in all three groups were done in duplicates, and the average OD values (9 values per group) were obtained (Matalon et al. 2009; Chen et al. 2020).

*Scanning electron microscopy (SEM):* was used to observe the morphology of the biofilms on the tested samples.

*Preparation of biofilm* (Chen et al. 2020).

Four disc-shaped specimens for each group were prepared similarly as the ADD and were placed in 24-well flat-bottomed tissue culture plates (HiMedia Pvt Ltd, Mumbai India) containing 2 ml BHI medium with 1% sucrose and *S. mutans* (10^8^ CFU/ml) in each well. After incubation for 24 h in a 5% CO_2_ incubator, a gentle rinse with 0.5% phosphate buffered saline (PBS) removed the non-adherent cells from the samples.

*SEM observation* Gram staining the smear obtained by scraping one disc in each group confirmed biofilm formation. The remaining three samples in each group went through the SEM observation for further exploratory analysis of the *S. mutans* biofilm. The samples were fixed with 2.5% glutaraldehyde and dehydrated through ascending series of 50%, 70%, 85%, 90%, and 100% ethanol for 15 min each. After drying, the samples were mounted on aluminium stubs, coated with 6 nm gold film, and examined under SEM at 2000×, 5000×, and 10,000× magnification. The images were analysed qualitatively for the *S. mutans* biofilm.

*Measurement of bacterial cells and surface area* For quantitative analysis of the *S. mutans*, the Image J software (Version 1.50b-2015, National Institutes of Health, Bethesda, USA) was used. Grids of 100 µm^2^ were drawn on the 10,000× SEM images. In each image, two adjacent grids (100 µm^2^ each) with high cell numbers were selected based on visual assessment. The bacterial cells were counted in the two grids using the measuring tool of the software, expressed as the number of cells per 200 µm^2^ surface of the specimen (Rahim and Thurairajah, 2011). The surface area occupied by bacteria per 200 μm^2^ was calculated using the magic wand tool of the software.

### Physical properties

### Net setting time

The stainless-steel rectangular moulds of dimensions (10 × 8 × 5 mm) and rounded internal corners, per ADA specifications were filled with mixed cement according to the mixing protocols described earlier. Mixing time was maintained between 15 and 20 s per manufacturer (GC Fuji IX Gold Label, GC Corporation Tokyo, Japan). Sixty seconds after the end of mixing, the unset specimens were indented with a Gilmore needle apparatus [needle mass 400 g, length 5 mm, and tip diameter 1 mm, per ANSI/ADA specification No. 96 (ISO 9917) at room temperature (30 °C)]. For a trial run to determine the approximate setting time, 90 s after the end of mixing, the indentations were repeated at 30-s intervals until the needle failed to make a complete circular indentation in the cement. Indentations were viewed at 2 × magnification using a magnifying lens. The needle was cleaned between indentations. To determine the net setting time, the indentations began 30 s before the approximate setting time, as defined by the trial run, making indentations at 10s intervals. The time was recorded using a stopwatch as the time elapsed from the end of mixing until the needle failed to make a complete circular indentation in the cement. Two independent examiners blinded to the group allocation repeated the test thrice in each group.

### Surface microhardness

Nine samples were prepared for each group as described earlier using brass moulds, 10 mm diameter, and 2 mm depth. To evaluate microhardness, the surface of each specimen was divided into four quadrants using a #11 scalpel blade. Vickers hardness number (VHN) was measured with a Digital Micro-Hardness tester (Matsuzawa MMT-X, Toshima Japan) using a 50gf load for 30 s. Two indentations were made in each quadrant with a 100 μm distance between them (visualised using a magnifying lens), giving eight values for each specimen, from which the mean was obtained (Marti et al. 2014). The investigator who conducted the assessment was blinded to the experimental groups.

### Compressive strength

Five specimens were prepared in each group, using brass split moulds, 4 mm diameter and 6 mm depth, per ANSI/ADA specification No. 96 (ISO 9917). One kg weight was placed on the top of the mould for one hour to standardise the pressure during the setting of the material, and the excess material was allowed to flow out. Twenty-four hours after mixing, an investigator unaware of the groups estimated the compressive strength using the universal testing machine (Instron 3366, Massachusetts USA). A compressive load at a crosshead speed of 0.75 mm/min was applied along the long axis. The compressive strength in megapascals (MPa) was calculated based on the maximum force applied and the surface area of the specimen.

### Statistical analysis

Data were analysed using IBM SPSS Statistics for Windows, Version 23 (IBM Corp., Armonk, NY, USA). Descriptive statistics were tabulated, and mean values were obtained. The test for normality (Shapiro–Wilk test) was not significant, and hence, all analysis was done using parametric tests. One-way ANOVA with post hoc Tamhane’s T2 test was applied to analyse the difference in net setting time, surface microhardness, compressive strength values, and the *S. mutans* count between the groups. As the control group values were zero, the Student’s *t* test was used to analyse the difference in the inhibition zones obtained by ADD. Repeated-measures ANOVA was used to check the difference in the OD values at different time intervals between the groups. For all the tests, the significance level was at a 95% confidence interval (*p < *0.05).

## Results

### Antibacterial activity

#### ADD

The C group showed no zone of inhibition at both 24 and 48 h after incubation. The test groups had inhibition zones at 24 and 48 h. The G1 group showed a mean inhibition zone of 8.90 ± 0.15 mm at both time intervals, while the G2 group showed a mean inhibition zone of 11.81 ± 0.18 mm at both intervals. Inhibition zone diameter ranged from 8.6 ± 0.15 to 9.10 ± 0.15 mm in the G1 group and from 11.60 ± 0.18 mm to 12.10 ± 0.18 mm in the G2 group. The G2 group had a higher diameter of inhibition zones, and the difference was statistically significant (*n* = 9; *t* = 178.00; *p < *0.001).

#### DCT

All groups except PC showed a decrease in the mean OD_650_ from 3 to 6 and 6 to 24 h (Table 1). NC had values at similar levels as group C, while groups C, G1, and G2 had lower OD_650_ values over time due to lower bacterial loads—the least decrease in C, followed by G1 and G2. OD_650_ readings in the PC were at a lower level for all periods. There was a significant between-group difference (Table 1), and Groups C and G2, C, and PC differed statistically in post hoc tests (*p = *0.025 and < 0.001, respectively) (Table 2). PC also differed significantly from G1, G2, and NC (*p < *0.001).

**Table 1:** Comparison of mean optical density (OD_650_) values measured by the direct contact test for *S. mutans* using Repeated-Measures ANOVA

Groups	*n*	Time			*F*	*p*-value
		3 h	6 h	24 h		
		Mean ± SD				
C	9	1.84 ± 0.09	1.83 ± 0.09	1.82 ± 0.10	54.61	< 0.001*
G1	9	1.79 ± 0.12	1.77 ± 0.13	1.55 ± 0.29		
G2	9	1.72 ± 0.09	1.46 ± 0.36	1.22 ± 0.42		
PC	4	0.32 ± 0.29	0.59 ± 0.10	0.39 ± 0.02		
NC	4	1.82 ± 0.14	1.80 ± 0.14	1.76 ± 0.17		

*SD* standard deviation, *C* control group, *G1* group 1 (Nisin 1%), *G2* group 2 (Nisin 3%), *PC* positive control, *NC* negative control. ^*^p < 0.05 significant

**Table 2:** Post hoc results of intergroup comparison of the mean optical density (OD650) values

Groups compared		Mean difference	*p*-value	95% Confidence interval (Lower–Upper)
C	G1	0.13	0.120	0.02–0.27
	G2	0.36	0.003*	0.10–0.62
	PC	1.66	< 0.001*	1.58–1.74
	NC	0.33	1.000	0.42–0.49
G1	G2	0.24	0.124	0.04–0.51
	PC	1.53	< 0.001*	1.41–1.66
	NC	0.09	0.980	0.49–0.31
G2	PC	1.30	< 0.001*	1.04–1.55
	NC	0.33	0.102	0.70–0.44
PC	NC	1.63	0.002*	1.09–2.17

*C* control group, *G1* group 1 (Nisin 1%), *G2* group 2 (Nisin 3%), *PC* positive control, *NC* negative control. ^*^p < 0.05 significant

#### Scanning electron microscopy (SEM)

##### Qualitative description.

**Fig. 1:**
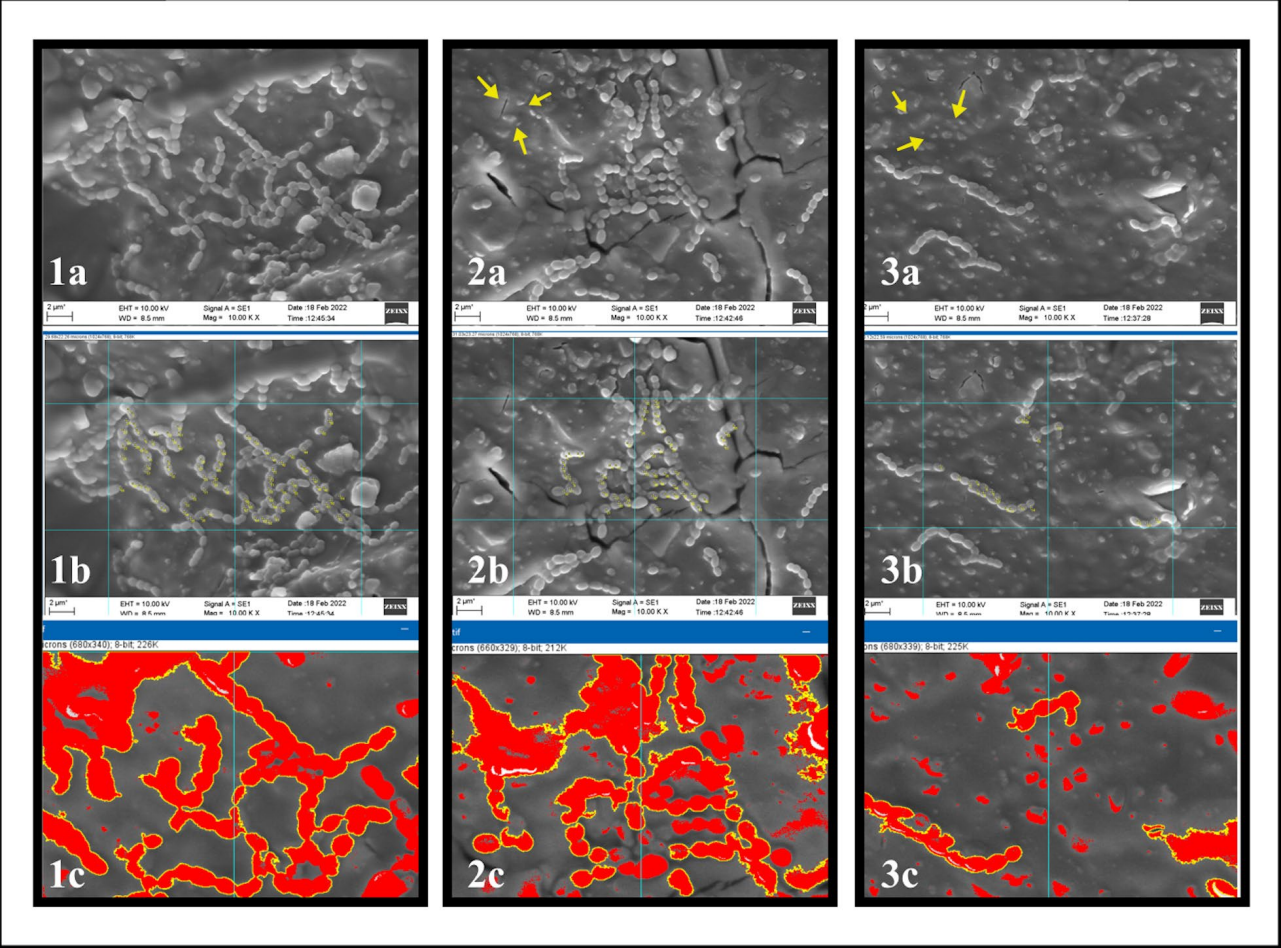
SEM image of the groups C(1a), G1(2a), and G2(3a) at 10,000 × magnification; yellow arrows indicate nisin particles in 2a and 2b. 1b, 2b, and 3b show the grids using Image J software for bacterial cell count estimation of the groups C, G1, and G2, respectively. 1c, 2c, and 3c represent areas demarcated using the magic wand tool of Image J software for bacterial cell surface area estimation of the groups C, G1, and G2, respectively

SEM observation of the specimens at 10,000× magnification (Fig. 1) revealed denser *S. mutans* biofilm in group C (Fig. 1a) when compared to G1 and G2 (Fig. 1b, c). In G1 and G2, unlike C, scattered between the bacterial cells were irregular particles, suggesting nisin particles on the surface. These particles were more in G2 than G1, consistent with higher nisin concentrations in G2.

##### Quantitative analysis.

The mean *S. mutans* cell count per 200 μm^2^ and cell surface area occupied by *S. mutans* cells recorded in 200 μm^2^ area of SEM image at 10,000× magnification by the groups are shown in Table 3. Group C had the highest count, followed by G1. G2 had > 50% lower *S. mutans* count, with a statistically significant difference in the cell count between the groups (*p = *0.004) (Fig. 1). Post hoc analysis revealed a significant difference between C and G2 Groups (*p = *0.049).

**Table 3:** Intergroup comparison of mean *S. mutans* count and surface area in 200 μm^2^ area of SEM image at 10,000 × magnification with post hoc results

Variable	*n*	C	G1	G2	F	*p*-value
		Mean ± SD				
Bacterial cell count (n/200 μm^2^)	3	109.00 ± 2.65^a^	91.00 ± 22.61^a,b^	36 ± 17.35^b^	15.90	0.004*
Bacterial surface area (%/200μm^2^)	3	81.27 ± 9.19^a^	71.43 ± 12.00^a^	17.33 ± 6.29^b^	39.81	< 0.001*

^a^ and ^b^same alphabets in superscript denote no statistical difference between the groups during post hoc analysis. *SD* standard deviation, *C* control group, *G1* group 1 (Nisin 1%), *G2* group 2 (Nisin 3%), ^*^p < 0.05 significant

The area occupied by bacterial cells was also the lowest in the G2 Group (8%), and the difference was significant (*p < *0.001). Post hoc analysis revealed a significant difference in the area covered by bacterial cells between C and G2 (*p = *0.003), and G1 and G2 (*p = *0.018) (Table 3).

## Physical properties

### Net setting time

**Fig. 2:**
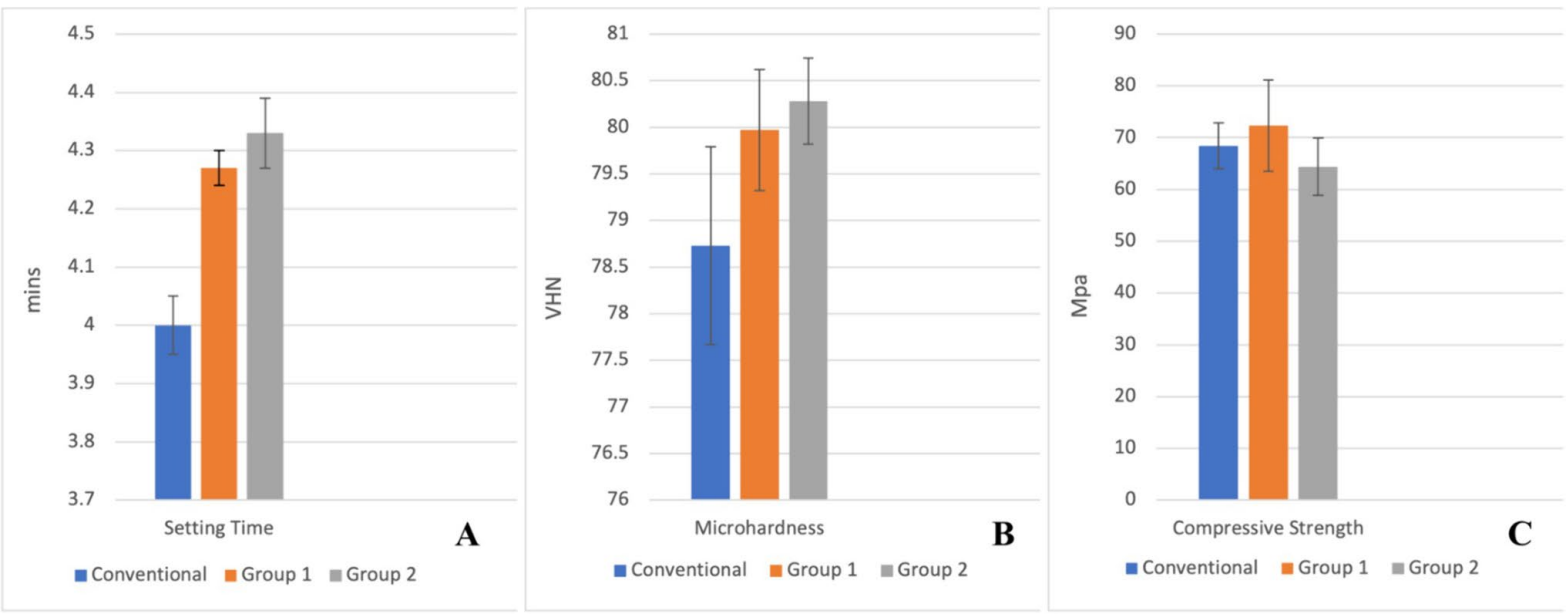
Column chart showing comparisons of mean net setting time **(A)**, surface microhardness **(B)**, and compressive strength **(C)** of the three groups

There was a statistically significant difference between groups (*n* = 3; *F* = 42.00; *p < *0.001) (Fig. 2). Post hoc analysis (Table 4) revealed a significant increase in the mean net setting time in G1 compared to the C group (*p = *0.010). The mean net setting time increased further in G2, and the difference was significant compared to C (*p = *0.010). There was no significant difference in the mean net setting times between G1 and G2 (Table 4).

**Table 4:** Post hoc results of intergroup comparison of mean net setting time and mean microhardness

Physical property	Groups compared		Mean difference	*p*-value	95% Confidence interval (Lower–Upper)
Setting time	C	G1	0.27	0.010*	0.11–0.42
		G2	0.33	0.010*	0.16–0.51
	G1	G2	0.07	0.440	0.12–0.25
Surface microhardness	C	G1	1.25	0.030*	0.92–2.38
		G2	1.56	0.010*	2.65–3.75
	G1	G2	0.31	0.610	1.03–3.28

*C* control group, *G1* group 1 (Nisin 1%), *G2* group 2 (Nisin 3%), ^*^p < 0.05 significant

### Surface microhardness

There was a statistically significant difference between the mean microhardness values of the groups (*n* = 9; *F* = 10.38; *p = *0.001) (Fig. 2). Post hoc analysis revealed a statistically significant difference in the microhardness between G2 and C (*p = *0.029), and between G1 and C (*p = *0.006), with G1 and G2 showing higher values than C. A dose-dependent increase in surface microhardness was observed, with no significant difference in the hardness between G1 and G2 (Table 4).

### Compressive strength

G1 had the highest value, followed by C and G2, but the difference was not statistically significant (Fig. 2) (*n* = 5; *p = *0.199).

## Discussion

The results show that the addition of 3% nisin to HVGIC significantly improved the antibacterial activity while significantly increasing the net setting time and the surface microhardness and not significantly affecting the compressive strength.

In our study, *S. mutans* was used to assess the antibacterial activity of nisin-modified HVGIC, as it is one of the primary microorganisms responsible for initiating dental caries, including secondary caries (Mjör and Toffenetti 2000). 1% and 3% concentrations of nisin were chosen to be added to HVGIC, based on earlier studies involving dental adhesives. Su et al. (2018) used 1%, 3%, and 5% nisin and found that all the concentrations improved antibacterial activity against *S. mutans*. The bond strength was affected at 3% and 5% concentration, while the degree of conversion was not affected. The antibacterial effects were concentration dependent. Lopes et al (2022) also found that 1%, 3% and 5% nisin addition to the etch and bond dental adhesive showed dose-dependent enhanced antibacterial activity, and at 1% concentration the degree of conversion and bond strength were not compromised. Zhao et al. (2020) used 1%, 2%, and 3% concentrations of nisin, the inhibitory effect on *S. mutans* was concentration dependent, with 3% showing the highest inhibition. At 3% concentration, multi-species biofilm was also inhibited. At all the concentrations, there was no significant effect on the bond strength and the degree of conversion. Thus, at 1 and 3% concentrations, the antibacterial activity against mono-species biofilms (*S. mutans*) was shown, without significantly affecting the bonding properties and degree of conversion of the material (Su et al. 2018; Zhao et al. 2020). At these concentrations, nisin is well above the MIC and MBC levels but within the levels of daily consumption of nisin in the diet or levels approved by the FDA (Cotter et al. 2005; Shin et al. 2016). Thus, 1% and 3%, which were above the MBC levels, were used in the current study, assuming that at these levels, the antibacterial effects of the HVGIC would be enhanced without altering the studied physical properties.

Group C had no zone of inhibition in the ADD test, implying that HVGIC has no antimicrobial action against *S. mutans,* per earlier evidence (Yesilyurt et al. 2009; Prabhakar et al. 2013). The antibacterial effect of HVGIC is due to fluoride release, which occurs by two mechanisms, burst release during setting and slow release from the set cement over time. However, the concentration of fluoride required for bacterial inhibition is much higher than the fluoride level released from the GIC (Wiegand et al. 2007). *S. mutans* were inhibited in both G1 and G2 groups, but the values did not increase after 24 h, implying no further effect. The result of ADD differed from that of nisin-modified dental adhesive, which showed no zone of inhibition at 24 h (Su et al. 2018). The release of nisin, as determined by ADD, can be attributed to the release of unbound nisin in the first 24 h, similar to chlorhexidine incorporated GIC (Yesilyurt et al. 2009; Bellis et al. 2016).

Ions, such as aluminium, calcium, silicon, and fluoride, are known to leach from GIC when in contact with an aqueous medium, contributing to OD values (Bapna and Mueller 1994). While NC represents OD values due to the ion leaching process, OD values of PC represent the typical growth pattern of bacteria in a culture medium that consists of initial exponential growth, followed by a plateau and then a decrease. Thus, the control and test group OD values represent turbidity due to both material ion leaching and bacterial growth. Assuming the turbidity due to the ion leaching process will be constant in all the groups, the OD values can represent antibacterial activity (Matalon et al. 2009). In an earlier study (Davidovich et al. 2007), DCT showed complete inhibition of *S. mutans* growth by GIC, which can be attributed to the burst of fluoride release immediately after the initial set of the cement, as the samples were inoculated immediately after sample preparation. In our study, the inoculation began 4 h after coating the unset cement in the microwells, and after disinfection in the UV chamber, giving contrast results. Our study noted a dose-dependent decrease in the mean OD_650_ values, a trend observed in the previous studies, where OD values for nisin-modified dental adhesives decreased dose-dependently (Su et al. 2018; Zhao et al. 2020; Keerthipriya et al. 2021).

SEM image analysis revealed nisin particles on the surface of HVGIC, as seen in an earlier study (Tong et al. 2011), which may have contributed to the surface antibacterial properties of the nisin-modified HVGIC. The nisin particles on the surface of the HVGIC can cause cell membrane damage via pore formation that causes ion leakage and ATP hydrolysis, leading to a bacteriostatic effect and limiting biofilm formation (Tong et al. 2011). The significant reduction in cell numbers and cell surface area of *S. mutans* in G1 and G2 further reinforces the antibacterial activity of nisin.

There was approximately a 30 s increase in the net setting time of groups G1 and G2 compared to group C. However, the values were well within the 2–6-min limit for GIC, per ANSI/ADA specification No. 96 (ISO 9917). Other antibacterial HVGIC modifications have increased net setting times dose-dependently (Prabhakar et al. 2013; Marti et al. 2014) due to decreased p/l ratio (Panpisut et al. 2021). The addition of nisin decreases the relative concentration of the polyacrylic acid in the liquid, reducing the availability of acidic groups for setting reaction (Nicholson 2018).

Groups G1 and G2 had significantly higher mean surface microhardness values than Group C, with no significant effects on compressive strength. However, there was a slight increase in the mean value of G1, though not statistically significant, as compared to other groups. The observed improvement in compressive strength and significantly better surface microhardness could be attributed to the reduced steric hindrance during the reaction between polyacrylic acid and glass powder. This facilitation is achieved by the addition of the amino acid peptide nisin, a phenomenon observed in other studies where amino acids were incorporated into GICs (Xie et al. 2006; Moshaverinia et al. 2009). As nisin is a lantibiotic with amino acids (Cotter et al. 2005; Chan et al. 2023), the effects are comparable with the addition of proline (an amino acid) to HVGIC, which increased surface microhardness at 24 h (Moshaverinia et al. 2009), and addition of amino acid acrylate or methacrylate derivatives to GIC that resulted in the increased flexural strength with no significant change in the compressive strength and diametral tensile strength (Xie et al. 2006). The amino acid’s polymeric structure acts as a spacer, reducing steric hindrance and increasing the carboxylic acid group mobility of GIC's polyacrylic acid. Polyacrylic acid then reacts more freely with calcium and aluminium ions of the powder, creating homogenous polysalt bridges that improve the modified material's mechanical properties (Xie et al. 2006; Moshaverinia et al. 2009; Ansari et al. 2013). Further, the amide groups present in nisin, like proline, may be capable of forming hydrophilic domains that cause increased inter and intra-molecular forces within the GIC matrix (Ansari et al. 2013). Interestingly, our study results diverge from findings in research involving other antibacterial modifications, such as antibiotics or chlorhexidine (Yesilyurt et al. 2009; Prabhakar et al. 2013; Tüzüner et al. 2019). Unlike those studies, we did not observe a dose-dependent reduction in mean compressive strength values for the experimental HVGICs compared to the unmodified control. Nisin is active at acidic pH (Chan et al. 2023), a property that facilitates the acid–base reaction of the glass ionomer. In contrast, other organic antimicrobial additives like chlorhexidine are alkaline and tend to form cationic salts with polyacrylic acid. This interaction interferes with the setting reaction between the fluoroaluminosilicate glass and polyacrylic acid in the glass-ionomer cement (GIC) (Marti et al. 2014).

The limitations of this study include short-term assessment of antibacterial and physical properties, no quantification of nisin release, and antibacterial property studied against single-species biofilm. Further in vitro research is required to study the long-term effects of the addition of nisin on various physical properties, including microleakage, bond strength, and flexure/tensile strength. As the bactericidal action of nisin appears to be concentration dependent, furthermore extensive studies are required to test the effect of the addition of nisin to HVGIC. Recognizing that the use of syringe technique such as Centrix syringe for material insertion into the moulds during specimen preparation can minimize variation between the samples, future experiments can consider the use of the syringe technique for material insertion during specimen preparation. Finally, clinical studies are needed to yield better evidence of the antibacterial activity and effectiveness of nisin-modified HVGIC. Considering that this is the first study to evaluate the effects of the addition of nisin into GIC, the findings of this study will serve as preliminary results for further research.

## Conclusion

Within limitations, the results of the study imply that the addition of 3% nisin (2.5% w/w pure) to HVGIC enhances the antibacterial action against *S. mutans* and surface microhardness without adversely affecting setting time and compressive strength.

## Data Availability

The data that support the findings of this study are available from the corresponding author upon reasonable request.
